# Impact of COVID-19 Pandemic on the Quality of Life of IBD Patients

**DOI:** 10.3390/medicina58050562

**Published:** 2022-04-19

**Authors:** Otilia Gavrilescu, Cristina Cijevschi Prelipcean, Mihaela Dranga, Iolanda Valentina Popa, Cătălina Mihai

**Affiliations:** 1 Medicale I Department, “Grigore T. Popa” University of Medicine and Pharmacy, 700115 Iasi, Romania; otilianedelciuc@yahoo.com (O.G.); catalinamihai@yahoo.com (C.M.); 2 “Saint Spiridon” County Hospital, 700111 Iasi, Romania; cristinacijevschi@yahoo.com; 3 Medicale II Department “Grigore T. Popa”, University of Medicine and Pharmacy, 700115 Iasi, Romania; iolivp@gmail.com

**Keywords:** COVID-19 pandemic, inflammatory bowel disease, quality of life

## Abstract

*Background and Objectives*: The COVID-19 pandemic has had a considerable impact on inflammatory bowel disease (IBD) patients by limiting their access to medical services due to restrictions and the reorganization of the healthcare systems, which affects their quality of life (QoL). We aimed to assess the impact of the COVID-19 pandemic on the QoL of patients with IBD. *Materials and Methods*: We conducted a descriptive observational study, which included 90 adult patients diagnosed with IBD. The study sample consisted of two subgroups: a retrospective-pre-pandemic group (group A) and a prospective-pandemic group (group B). Group A included 45 IBD patients who were evaluated in 2018. Group B included 45 patients with confirmed diagnosis of IBD, evaluated between June and December 2021—the period of the COVID-19 pandemic (prospective), consecutively recruited. All the patients filled in a QoL assessment questionnaire—IBDQ-32. Subsequently, the two samples were comparatively assessed. *Results*: The average values of the IBDQ scores were significantly lower in 2021 compared to those recorded in 2018: 145.56 vs. 128.3 (*p* < 0.05). We also we found significant differences between the subscores: IBDQ1 (*p* = 0.043), IBDQ2 (*p* = 0.034), IBDQ3 (*p* = 0.045), IBDQ4 (*p* = 0.025). *Conclusions*: IBDQ scores were significantly lower in 2021 compared to 2018 (*p* < 0.05), showing that during the COVID-19 pandemic, patients with IBD had a more influenced QoL.

## 1. Introduction

The COVID-19 pandemic is an ongoing global problem caused by the new SARS-CoV-2 coronavirus strain, first identified in Wuhan in December 2019. In January 2020, the World Health Organization stated that the outbreak constituted a public health emergency of international concern and, in March 2020, declared the COVID-19 pandemic [1]. By February 2022, more than 400 million cases had been confirmed, with more than 229 million recoveries and over 5 million deaths [2], turning it into one of the deadliest pandemics in history. The COVID-19 mortality is estimated to be as high as 1.5% to 3.8% [3,4]. COVID-19 has created major dysfunctions in the healthcare systems worldwide, with consequences especially among patients with chronic diseases [5], including patients with inflammatory bowel diseases (IBD)—Crohn’s disease (CD) and ulcerative colitis (UC) [6]. Inflammatory bowel diseases are chronic inflammatory relapsing–remitting diseases affecting the gastrointestinal tract and requiring immunosuppressive or immunomodulator therapies for induction and maintenance of remission [6]. IBD patients require repeated hospital visits for follow-up, injectable therapies, investigations, and surveillance endoscopies, which can increase exposure to COVID-19 infection.

In the context of the pandemic, patient monitoring has suffered due to restrictions and the reorganization of the healthcare systems. The cancellation of scheduled monitoring visits and their replacement with “digital visits” and the interruption of or delay in the application of specific medication (biologics, immunosuppressive therapies) are likely to increase the morbidity and mortality among these patients in the medium and long term [7]. Furthermore, IBD-specific immunosuppressive/immunomodulatory medication may lead to higher susceptibility to SARS-CoV-2 infection or to a more severe course of the disease [7]. Therefore, IBD patients fall within the fragile, at-risk population vulnerable to the SARS-CoV-2 infection.

Nevertheless, there is currently no data to suggest an increased risk of infection or a more severe course of SARS-CoV-2 infection in patients with IBD [6]. Studies assessing the characteristics, evolution, and risk factors associated with severe forms of COVID-19 infection in patients with IBD have reported issues similar to those of the general population [7,8]. However, most of these studies are limited by the small number of patients [9]. Despite their immunosuppressed status and specific medication, IBD patients seem to develop mild forms of COVID-19 [10,11]. This may be due to the immunosuppression, which is likely to protect patients against developing an uncontrolled immune response to SARS-CoV-2 [12].

The COVID-19 pandemic has had a considerable impact on the chronically ill, primarily by limiting their access to medical services, but also by the inherent mental burden [13]. The fear of SARS-CoV-2 infection, physical distancing, isolation, loneliness, and the uncertain future have negatively affected quality of life (QoL) [13,14]. The actual extent of the effects of the pandemic is yet to be known. The results of a study evaluating the impact of epidemiological measures on social distancing and isolation at home showed that symptoms of anxiety and depression, as well as QoL impairment, were found in 20–30% of patients [15]. Other studies that quantified the effects of COVID-19 epidemiological measures on the health of patients with chronic conditions showed that QoL was unaffected by COVID-19 [16,17]. Based on these premises, the present study aims to assess the impact of the COVID-19 pandemic on the QoL of patients with IBD.

## 2. Materials and Methods

We conducted a descriptive observational study, which included 90 adult patients diagnosed with IBD. The study group consisted of two subgroups: a retrospective-pre-pandemic group (group A) and a prospective-pandemic group (group B). Group A included 45 IBD patients who were evaluated in 2018 as part of a grant project—“*The specialized educational and psychological counselling in inflammatory bowel disease patients*” [18]. Group B included 45 patients with confirmed diagnosis of IBD, evaluated at the Institute of Gastroenterology and Hepatology in Iasi, between June and December 2021—the period of the COVID-19 pandemic (prospective), consecutively recruited. Medical reports of patients included in the study group were retrieved from the Saint Spiridon Country Hospital, Iasi database. The IBD diagnosis was established by colonoscopy and histopathological examination. In particular cases, additional examinations were required (small bowel capsule endoscopy, computed tomography enterography, and magnetic resonance enterography). Patients with nonspecific colitis, *Clostridioides difficile* infection, colon cancer, and patients with known psychiatric disorders were excluded from the study. A fact sheet including the following data was prepared: type of disease (UC/CD), age, sex, physical environment, disease activity (Ulcerative Colitis Activity Index—UCDAI and Crohn Disease Activity Index—CDAI), extent and disease phenotype (Montreal classification) and type of treatment: biologicals (e.g., infliximab, adalimumab, vedolizumab, ustekinumab), tofacitinib, corticosteroids (e.g., prednis(ol)one, budesonide), immunosuppressants (e.g., azathioprine) and mesalamines (e.g., sulfasalazine and mesalazine).The clinical activity was evaluated according to the Crohn’s disease activity index (CDAI) for patients with CD. A score of less than 150 corresponds to remission; 150 to 219, mild disease; 220 to 450, moderately active disease; and greater than 450, severe disease. For patientswith UC the clinical activity was evaluated according to the Ulcerative Colitis activity index (UCDAI). A score less than 2 corresponds to remission; 3 to 5, mild disease; 6 to 8, moderately active disease; and 9–12, severe disease.

All the patients filled in a QoL assessment questionnaire—IBDQ-32 (Inflammatory Bowel Disease Questionnaire-32). The IBDQ is a disease-specific QoL questionnaire. It consists of 32 items divided into four domains: bowel-related symptoms, systemic symptoms, emotional function, and social function. Responses are graded on a seven-point scale in which 1 indicates the worst function and 7 the best function. The total IBDQ score gives a possible range of 32–224, with a higher score indicating a better QoL [19]. Subsequently, the two groups were comparatively assessed. 

### 2.1. Statistical Analysis

The data were systematized and centralized in an SPSS 18.0 database and was processed with adequate statistical functions. In presenting the data, we used 95% confidence intervals. Primary indicators (minimum, maximum, frequency), average value indicators (mean, median, modulus), and dispersion indicators (standard deviation, standard error, mean confidence interval) were used for the descriptive statistical analysis. The Skewness test (−2 < *p* < 2) was used to verify the homogeneity of the series of values.

To compare the variables from the two years of study, we relied on qualitative significance tests, such as the chi2 test, which compares two or more frequency distributions. The T-Student test takes into account the measurement of variability and the weight of observations, based on the mean and standard deviation. For each analyzed group, a “t” is calculated, depending on the number of degrees of freedom (df). The F ANOVA test measures the difference in the mean values from three or more groups.

### 2.2. Ethical Aspects

All patients enrolled in the study signed an informed consent, which explained details about the purpose of the study, its methodology, and the risks and benefits involved in the study, as well as information regarding the confidentiality of the results. The study was conducted according to the Declaration of Helsinki and approved by the Ethic Committee of “Grigore T. Popa” University of Medicine and Pharmacy in Iasi (code 112, 4 October 2021).

## 3. Results

Most patients enrolled in the study were male (64.4%), over the age of 45 (51.1%), and from urban areas (73.3%) (Table 1).

The proportion of patients with UC was significantly higher compared to that of patients with CD (69% vs. 31%). Nevertheless, in the 2021 group of patients with CD, there was an increase in the number of CD cases compared to 2018 (37.8% vs. 24.4%).

**Figure 1:**
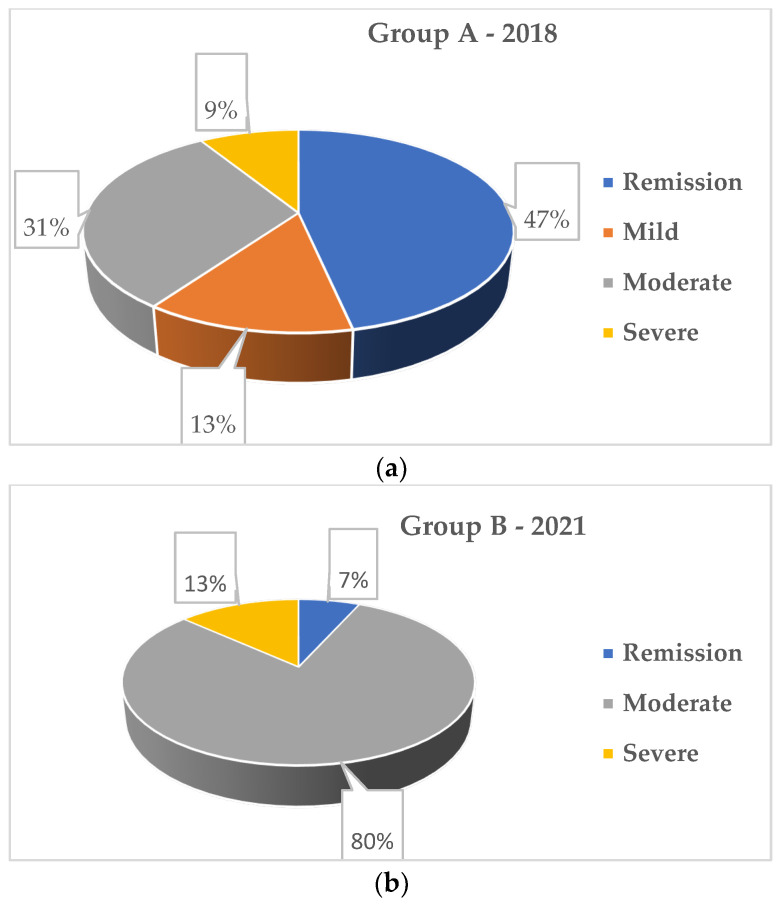
(**a**) Disease activity—group A (2018) (**b**) Disease activity—group B (2021).

In group A, before the pandemic, 46.7% of the patients were in remission; moderate IBD activity was found in 31.3% of cases, while mild and severe activity was present in smaller proportions (13.3% and 8.9%). In group B, during the pandemic, most patients had moderate disease activity (80%; *p* = 0.001). Patients with mild disease activity were not found in this group, and those with severe activity were found in a higher proportion compared to group A (8.9% vs. 13.3%). The percentage of patients with CD in remission was significantly lower compared to group A (6.7% vs. 46.7%) (*p* = 0.001) (Figure 1, Table 1). Only two patients from group B had a history of mild SARS-CoV-2 infection, and none of the patients had any active SARS-CoV-2 infection documented at the time of the study. None of the patients underwent a surgical procedure before or during the study period.

Regarding the QoL of IBD patients, the average values of the IBDQ scores were significantly lower in 2021 compared to those recorded in 2018: 145.56 vs. 128.3 (*p* < 0.05) (Table 2). Equally, the mean values of the IBDQ-1 and IBDQ-2 subscales assessing the impact of gastrointestinal and systemic symptoms on QoL were 43.24, respectively 20.67. The mean values of the IBDQ-3 and IBDQ-4 subscales assessing the emotional function and the social function were 45.2 and 19.22, respectively. We applied linear regression to adjust for disease activity as a confounding factor. We showed that the impact of the pandemic remains significant even after adjustment. The linear regression coefficients (illustrated in Table 2) prove that both the pandemic and the disease activity have an independent significant impact on IBDQ.

Correlating the IBDQ scores with the disease activity, we noticed that in group A, the lowest values of IBDQ scores were found in patients with severe IBD activity, but without statistically significant differences (*p* > 0.05). In group B, the patients with severe IBD activity had significantly lower IBDQ scores compared with the patients with moderate disease activity (*p* < 0.001).

Considering that the IBD activity represents one of the most important factors influencing QoL, we contrasted the values of QoL scores from 2018 (before the pandemic) and 2021 (during the pandemic). We found a significant decrease in the QoL scores in patients with severe activity from group B (the pandemic group) (Table 3).

## 4. Discussion

The emergence of the COVID-19 pandemic constitutes a new event with devastating effects around the world, which has led to changes in human behavior, lifestyle, and the reality in which we live. The pandemic has caused feelings of uncertainty, along with isolation, anxiety, and sadness [20].

It is well known that stressful events and chronic stress are factors that can contribute to the increase in psychological disorders, the impairment of QoL, and even the onset of disease activity in patients with IBD [21]. 

The epidemiological measures imposed during the pandemic have significantly reduced patients’ communication and relationships with healthcare professionals. Thus, in addition to the anxiety caused by the risk of infection with the COVID-19 virus, patients have also been deprived of the psychological and medical support they were used to [22]. Recently, several studies have evaluated the effects of COVID-19 on the health of patients with chronic disorders [23]. The relationship between chronic organic and psychological disorders is a bidirectional one. On the one hand, psychological stress can lead to the decompensation of organic diseases by neuroendocrine and inflammatory mechanisms [24] and. On the other hand, chronic disease can be a stressor that activates subjective stress and excessive health concerns by allostatic overload [25]. 

Although the relationship between emotional stress and gastrointestinal symptoms requires more evidence, the present study provides an additional argument in this direction. In the groups considered, IBDQ scores were significantly lower in 2021 compared to 2018. The surveyor who assessed the impact of gastrointestinal symptoms on QoL (IBDQ-1) showed significantly lower mean values during the pandemic (49.51 vs. 43.24; *p* = 0.043). Equally, mean baseline values assessing the impact of systemic symptoms on QoL (IBDQ-2) were significantly lower during the pandemic (23.84 vs. 20.67; *p* = 0.034). According to the results of this study, patients with IBD showed more severe gastrointestinal and systemic symptoms during the pandemic–symptoms that significantly influenced the patients’ QoL. 

The results of our study support data from the literature [26,27]. A recent study evaluating the effects of social isolation and emotional stress on patients with IBD during the COVID-19 pandemic showed that isolation at home was associated with an increase in the frequency of stress episodes (50.8%) and gastrointestinal, predominantly functional symptoms, while the decrease in the frequency of stress episodes (11.1%) was associated with a decrease in gastrointestinal symptoms [28]. Among all the evaluated stressors, the aggravation of the gastrointestinal functional symptoms was mainly related to the accentuation of the interpersonal tensions and the excessive interpersonal proximity [28]. Periods of loneliness have been associated with the onset of IBD outbreaks, gastrointestinal functional clinical manifestations, and impaired subjective health. Finally, the anxiety associated with the COVID-19 pandemic was predominantly related to the severity of IBD symptoms and impairment of social life [28].

The first immediate effect of the pandemic was on social life, even affecting interpersonal relationships [29]. The severity of the infection with the new Corona virus, and especially its aggressive contagiousness causes feelings of concern and even anxiety, accentuating the collective state of panic and confusion [30]. A recent research showed that IBD patients had COVID-19 as frequently as non-IBD controls despite immunosuppressive therapy, possibly due to their awareness and preventive practices. Regarding the QoL scores, although the physical, psychological, and social QoL scores during the COVID-19 pandemic were comparable to the prepandemic period, the environmental scores were worse [31]. The COVID19 outbreakhad a negative impact on the QoL of IBD patients in remission, with higher self-perceived stress scores associated with a lower QoL [32].

In the present study, the mean values of the subscale assessing the impact of IBD on social life (IBDQ-3) were significantly lower during the pandemic (52.7 vs. 45.20; *p* = 0.045). Social isolation was hard to tolerate, the disappearance of many forms of leisure created feelings of “emptiness”, and maintaining a balanced mental state became a challenge.

The pandemic has been a completely new experience for everyone, which has induced a global, not just individual, state of mental discomfort [27]. In our study, the sub-scores assessing the impact of IBD on emotional life (IBDQ-4) showed significantly lower mean values during the pandemic (22.1 vs. 19.22; *p* = 0.025). The pandemic has caused many difficult emotions—insecurity, sadness, disappointment, anger, guilt, frustration, or loneliness. All this has had a major negative impact on QoL.

In conclusion, in the evaluated groups, IBDQ scores were significantly lower in 2021 compared to 2018 (*p* < 0.05), showing that during the COVID-19 pandemic, patients with IBD had a more influenced QoL.

The results of our study showed an increase in the number of cases of CD compared to 2018 (37.8% vs. 24.4%). These results confirm the data in the literature, according to which the frequency of CD has increased lately, pointing to a levelling tendency in the frequency of the two diseases [33].

Given that during the pandemic, in order to reduce the risk of infection with the SARS-CoV-2 virus, specialist medical visits were limited as much as possible, patients with mild disease activity are absent from the pandemic group. Most of the patients evaluated in this group presented moderate disease activity (80%), and those with severe activity were found in a higher proportion compared to the group before the pandemic (8.9% vs. 13.3%).

An increasing number of studies have shown that emotional stress is one of the major triggers for the symptoms of IBD patients [28,29]. The incidence of emotional disorders is higher among patients with IBD compared to the general population, and the presence of conditions such as depression and anxiety influence the evolution and severity of the disease [34,35].

Our study has several limitations. Firstly, the disease activity was significantly different between the two groups of patients, thus introducing an important confounding factor. This limitation was due to the fact that during the pandemic, the IBD patients who went to the hospital generally presented moderate-severe forms of the disease, with clinical manifestations that could not be managed at home. However, we adjusted our analysis to control for the confounding factor using linear regression, thus significantly reducing the risk of bias. Another limitation of the study consists in the small number of patients, which was due to the fact that the first group was evaluated as part of a research grant project [18]. Therefore, the number of patients included in the study was limited both by funding available and the project structure. Consequently, a similar number of patients had to be selected for the pandemic group for comparison. Therefore, further longitudinal studies have to be conducted in order to assess the long-term impact of the COVID-19 pandemic on QoL, the psychological state, as well as the evolution of the disease in IBD patients.

## 5. Conclusions

To conclude, the present study demonstrates the changes in the QoL of IBD patients, with all underlying implications, irrespective of the disease activity. This research provides a comparative assessment of QoL in IBD patients before and after the pandemic, a topic which has been relatively little explored in the literature.

The COVID-19 pandemic has fundamentally changed the landmarks of the world we live in, with considerable effects on both physical and mental health. The pandemic has not only been an epidemiological or immunological problem, but it has also had a significant psychological impact [36].

This pandemic is not over yet, and the psychological consequences will continue to affect the chronically ill. Identifying and acknowledging the medium- and long-term effects of the pandemic is necessary to mitigate the negative impact and to be able to provide specialized support to these categories of patients.

## Figures and Tables

**Table 1:** Structure of the study group.

	Total *n* = 90	2018 Group *n* = 45	2021 Group *n* = 45	*p*
Mean age ± SD years (median/interval)	43.77 ± 12.76 44/19–70	43.80 ± 12.91 44/19–70	43.73 ± 12.75 44/19–70	0.980 ^t^
Male, *n*(%)	58 (64.4%)	27 (60.0%)	31 (68.9%)	0.583 ^C^
≥45 years, *n* (%)	46 (51.1%)	23 (51.1%)	23 (51.1%)	1.000 ^C^
Urban, *n* (%)	66 (73.3%)	31 (68.9%)	35 (77.8%)	0.339 ^C^
Disorder, *n* (%)				0.171 ^C^
UC	62 (68.9%)	34 (75.6%)	28 (62.2%)	
CD	28 (31.1%)	11 (24.4%)	17 (37.8%)	
Activity, *n* (%)				**0.001** ^C^
mild	6 (6.7%)	6 (13.3%)	0 (0.0%)	
moderate	50 (55.6%)	14 (31.3%)	36 (80.0%)	
severe	10 (11.1%)	4 (8.9%)	6 (13.3%)	
Remission, *n* (%)	24 (26.7%)	21 (46.7%)	3 (6.7%)	**0.001** ^C^
Mesalamines, *n* (%)	31 (34.4%)	16 (35.6%)	15 (33.3%)	0.825 ^C^
Immunosupressants, *n*(%)	19 (21.1%)	9 (20.0%)	10 (22.2%)	0.797 ^C^
Corticosteroids, *n* (%)	8 (8.9%)	3 (6.7%)	5 (11.1%)	0.461 ^C^
Biologicals, *n* (%)	32 (35.6%)	17 (37.8%)	15 (33.3%)	0.661 ^C^

C—Chi square test, t—Student (*t*) test. The *p*-values below 0.05 are marked in bold. UC: ulcerative colitis; CD: Crohn’s disease

**Table 2:** Mean values of the IBDQ scores compared by year of study.

Score	2018 Group *n* = 45	2021 Group *n* = 45	Statistical Test	*p*	Liniar Regression			
					Standardized Coefficients			Sig.
					(Constant)	Year	Activity	
IBDQ	145.56 ± 32.00	128.33 ± 34.76	t-Student	**0.016**	153.50	5.50	−18.50	**0.018**
IBDQ-1	49.51 ± 13.13	43.24 ± 15.76	t-Student	**0.043**	53.15	3.41	−8.49	**0.018**
IBDQ-2	23.84 ± 7.03	20.67 ± 6.95	t-Student	**0.034**	22.32	1.84	−2.78	0.090
IBDQ-3	52.07 ± 16.46	45.20 ± 15.62	t-Student	**0.045**	62.50	−3.44	−5.36	0.210
IBDQ-4	22.11 ± 5.93	19.22 ± 6.12	t-Student	**0.025**	27.05	0.63	−4.39	**0.003**

**Table 3:** Correlation of the IBDQ score with the disease activity by study group.

Score	Disease Activity			Statistical Test	*p*
	Mild	Moderate	Severe		
**2018 Sample**					
IBDQ	133.75 ± 8.44	122.43 ± 28.80	119.33 ± 24.99	F ANOVA	0.674
IBDQ-1	43.75 ± 3.95	37.43 ± 11.13	44.67 ± 14.01	F ANOVA	0.350
IBDQ-2	18.50 ± 1.73	18.57 ± 5.87	19.67 ± 6.74	F ANOVA	0.918
IBDQ-3	55.75 ± 10.34	49.64 ± 19.01	42.17 ± 18.97	F ANOVA	0.499
IBDQ-4	15.75 ± 4.03	19.79 ± 5.04	20.33 ± 6.86	F ANOVA	0.376
**2021 Sample**					
IBDQ	-	121.39 ± 24.21 *^p^* ^= 0.144^	67.67 ± 20.40 ***^p^*^= 0.001^**	F ANOVA	**0.001**
IBDQ-1	-	35.97 ± 13.23 *^p^* ^= 0.067^	22.67 ± 12.74 ***^p^*^= 0.014^**	F ANOVA	**0.001**
IBDQ-2	-	18.03 ± 6.63 *^p^* ^= 0.231^	14.17 ± 4.54 *^p^* ^= 0.110^	F ANOVA	**0.001**
IBDQ-3	-	48.25 ± 12.24 *^p^* ^= 0.760^	19.50 ± 10.35 ***^p^*^= 0.001^**	F ANOVA	**0.001**
IBDQ-4	-	18.14 ± 5.52 *^p^* ^= 0.836^	11.33 ± 4.08 *^p^* ^= 0.131^	F ANOVA	**0.001**

## Data Availability

The datasets generated during and/or analyzed during the current study are available from the corresponding author on reasonable request.
